# Healthcare Spending Before and After Mild Cognitive Impairment Diagnosis: Evidence from the NHIS–NHID in Korea

**DOI:** 10.3390/healthcare13162076

**Published:** 2025-08-21

**Authors:** Sujin Ma, Huiwon Jeon, Yoohun Noh, Jin-Won Noh

**Affiliations:** 1 Department of Health Administration, Graduate School of Yonsei University, Wonju 26493, Republic of Korea; sujin.ma0217@gmail.com; 2 Department of Health and Medical Information, Ansan University, Ansan 15328, Republic of Korea; hwjeon210@gmail.com; 3 Emocog Inc., Seoul 08708, Republic of Korea; 4 Division of Health Administration, College of Software and Digital Healthcare Convergence, Yonsei University, Wonju 26493, Republic of Korea

**Keywords:** mild cognitive impairment (MCI), healthcare expenditures, aging population, comorbidities, socioeconomic disparities

## Abstract

**Background/Objectives:** With rapid population aging, concerns about cognitive health—especially mild cognitive impairment (MCI), a prodromal stage of dementia—are growing. Although MCI prevalence is rising, limited empirical evidence exists on changes in healthcare expenditures associated with its diagnosis. This study aimed to assess shifts in medical spending before and after MCI diagnosis and to identify factors influencing healthcare costs among Korean adults. **Methods:** We used data from the National Health Insurance Service–National Health Information Database (NHIS–NHID) from 2020 to 2022. This study analyzed 4162 Korean adults aged ≤84 who were newly diagnosed with MCI in 2021. Annual healthcare expenditures were tracked from 2020 to 2022. Generalized estimating equations (GEEs) were employed to examine changes over time, adjusting for sociodemographic characteristics, comorbidities, healthcare utilization, and long-term care insurance (LTCI) enrollment. **Results:** The average annual healthcare expenditure increased from 74,767 KRW before diagnosis to 87,902 KRW after diagnosis, reflecting a 12.51% rise. Regression analysis showed a significant decrease in costs in the year prior to diagnosis (β = −0.117, *p* < 0.01) and an increase in the year following diagnosis (β = 0.061, *p* < 0.01). Higher expenditures were associated with greater outpatient visits (β = 0.385, *p* < 0.01), longer hospital stays (β = 0.039, *p* < 0.01), LTCI enrollment (non-graded: β = 0.035, *p* = 0.02; graded: β = 0.027, *p* = 0.04) and higher comorbidity levels (CCI = 2: β = 0.088, *p* < 0.01, CCI ≥ 3: β = 0.192, *p* < 0.01). Conversely, older age (β = −0.003, *p* = 0.02) and female sex (β = −0.093, *p* < 0.01) were associated with lower costs. Sex-stratified analyses revealed consistent cost trends but different predictors for male and female patients. **Conclusions:** Healthcare expenditures rise significantly after MCI diagnosis. Early identification and interventions tailored to patient characteristics—such as age, sex, and comorbidity status—may help manage future costs and support equitable care for older adults.

## 1. Introduction

The aging of the global population has contributed to a notable increase in cognitive health concerns, especially mild cognitive impairment (MCI) [1,2,3]. This condition is often regarded as an intermediate stage between age-related cognitive decline and the onset of dementia, marked by measurable cognitive deterioration that does not yet significantly hinder daily activities [1,4]. Compared to the general elderly population, where the estimated annual progression rate to dementia is around 1–2%, individuals diagnosed with MCI exhibit a notably higher risk, with rates ranging between 10% and 15% [5,6]. Given these trends, the early identification and effective clinical management of MCI have received growing interest in both research and health policy discussions [2,4,7].

South Korea, where demographic aging is occurring at one of the most accelerated rates globally, presents a compelling case. By 2025, projections suggest that over one-fifth of the population will be aged 65 years or older [8]. Epidemiological data from large-scale national studies indicate that approximately 22.6% of individuals in this age group meet criteria for MCI [3]. This high prevalence calls attention to the urgent need for targeted strategies aimed at managing the broader societal and healthcare burden posed by cognitive decline in older adults.

The financial burden associated with the diagnosis and treatment of MCI is considerable, primarily because cognitive decline is frequently accompanied by increased use of healthcare services. These include, but are not limited to, outpatient care, medication prescriptions, and hospital admissions [5,6,9]. In a study by Lin et al., Medicare spending was reported to rise notably following an MCI diagnosis, with the majority of this increase attributed to outpatient visits and pharmaceutical expenses [6]. Similarly, research conducted by Leibson et al. identified a marked escalation in medical expenditures among individuals with MCI, which was largely driven by greater rates of hospitalization and the clinical management of coexisting chronic illnesses [5]. These empirical observations highlight the importance of analyzing medical costs in relation to cognitive health status, as such insights are critical for the development of effective health policies and rational allocation of healthcare resources.

Although global interest in MCI has grown considerably, there remains a notable lack of empirical research specifically examining variations in healthcare expenditures before and after the clinical diagnosis of MCI, particularly in Korea. Existing studies in this area are frequently limited by small sample sizes or confined to specific clinical settings, which constrains the external validity and generalizability of their conclusions [7,9]. In this regard, the Korean National Health Insurance Service (NHIS) offers a comprehensive claims database that encompasses nearly the entire Korean population, thereby providing a robust foundation for population-level analyses of medical service utilization and cost patterns associated with cognitive decline [10]. Furthermore, while previous research has explored average cost trajectories related to MCI or dementia, few studies have conducted sex-stratified analyses using diagnosis-based cohorts particularly in Korea. Most existing literature adjusts for sex as a control variable, rather than examining gender-specific patterns in healthcare use and cost drivers. This study is among the first to address that gap by applying a stratified regression approach to assess how medical expenditures evolve differently by sex following MCI diagnosis [11]. Against this background, the present study seeks to assess changes in healthcare expenditures before and after the initial diagnosis of MCI among Korean adults, using data from the National Health Information Database (NHIS–NHID), maintained by the National Health Insurance Service. In addition to quantifying cost trajectories, the study seeks to identify sociodemographic and clinical factors—including sex-specific differences—that influence these expenditures. By employing sex-stratified regression model, we aim to uncover gender-based disparities in healthcare utilization following MCI diagnosis. These findings are expected to inform more equitable and targeted policy interventions. Ultimately, by analyzing nationally representative data, this study will provide robust empirical evidence to guide public health planning, clinical practice, and efficient resource allocation for managing cognitive impairment in aging populations.

## 2. Materials and Methods

### 2.1. Data and Study Population

**Figure 1:**
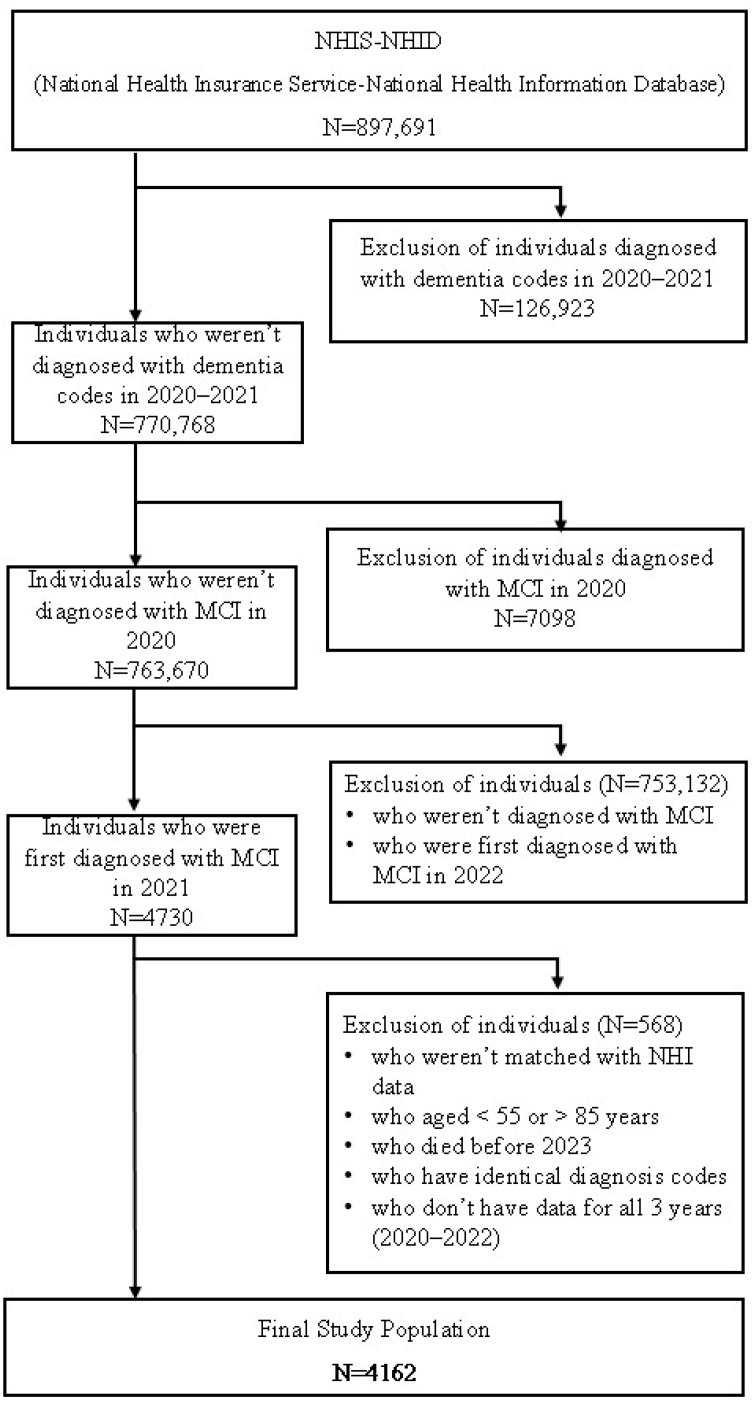
Study Population Inclusion/Exclusion Criteria Flow chart. (MCI: Mild Cognitive Impairment).

This study utilized claims data from the NHIS–NHID, spanning three years from 2020 to 2022. A total of 4162 Korean adults aged ≤84 years who were continuously enrolled in the database during the study period were included, based on the following eligibility criteria (Figure 1).

The study population consisted of individuals newly diagnosed with MCI in 2021, defined by an ICD-10 code of F067 as the primary diagnosis, with no record of a F067 diagnosis in 2020 (washout period). To reduce diagnostic overlap, individuals were excluded if any of their primary to quinary diagnoses included one or more of the following ICD-10 codes: F00 (Dementia in Alzheimer’s disease), F01 (Vascular dementia), F02 (Dementia in other diseases classified elsewhere), F03 (Unspecified dementia), G30 (Alzheimer’s disease), G31 (Other degenerative diseases of nervous system, not elsewhere classified), F20–F25 (Schizophrenia spectrum and other psychotic disorders). Furthermore, individuals who died before 2023 and those with identical diagnosis codes (e.g., primary diagnosis = secondary diagnosis, quaternary diagnosis = quinary diagnosis) were excluded from the study population in accordance with the mutual exclusivity principle of the ICD-10 classification.

### 2.2. Variables

The primary aim of this study was to assess changes in medical expenditures before and after a new diagnosis of MCI, as well as to identify factors associated with healthcare spending.

The key independent variable was time relative to the MCI diagnosis, categorized into three periods: pre-diagnosis (2020), diagnosis year (2021), and post-diagnosis (2022). Additional independent variables included the number of inpatient and outpatient visits and total length of stay. Socio-demographic and health-related covariates were also analyzed, including: sex; age; urbanicity (metropolitan, mid-sized city, rural); income level based on health insurance premium quintiles (low: 0–6, middle: 7–13, high: 14–20); disability status (non-disabled, mild disability, severe disability); health insurance type (medical aid, self-employed, employed); Charlson Comorbidity Index (CCI) score (0, 1, 2, ≥3); and long-term care insurance (LTCI) status (non-recipient, non-graded, graded).

The dependent variable was total medical expenditure, defined as the average annual healthcare cost per individual. Individuals with no recorded medical expenditures during the observation period were excluded from the final analysis. Also, in log-transformed models, the percentage change in medical expenditure can also be interpreted using the formula: 100 × (exp(β) − 1) [12].

### 2.3. Statistical Analysis

Descriptive statistics were used to summarize the demographic and clinical characteristics of the study population. A generalized estimating equation (GEE) model was employed to estimate longitudinal changes in healthcare costs, accounting for repeated measures within individuals over time. This approach is appropriately adjusted for within-subject correlation. A gamma distribution with a log link function was applied due to the right-skewed nature of medical expenditure data. We also performed a sensitivity analysis to minimize the influence of outliers. In the sensitivity analysis, we winsorized the total expenditure data at 99.9% and 0.1% percentile [13]. All statistical analyses were conducted using SAS version 9.4 (SAS Institute Inc., Cary, NC, USA). A *p*-value of < 0.05 was considered statistically significant. Expenditures are measured in Korean Won (KRW) and also converted to US dollars (USD), calculated at a currency exchange rate of $1 to 1356.00 KRW as of 2 July 2025.

### 2.4. Ethical Consideration

Informed consent for this study was waived by the Institutional Review Board (IRB) at Yonsei University Mirae Campus (approval no. 1041849-202307-SB-124-01) on 27 July 2023 due to the retrospective and anonymized nature of the data.

## 3. Results

### 3.1. Characteristics of the Study Population

Table 1 presents the baseline characteristics of the 4162 study participants. The majority were female (66.84%, n = 2782), with an average age of 70.59 years at the time of MCI diagnosis. Medium-sized cities were the most common residential setting (45.96%, n = 1913). Nearly half (48.32%, n = 2011) of the participants belonged to the low-income group, and a substantial proportion had significant comorbidities, with 40.12% (n = 1670) scoring ≥3 on the CCI. Most participants had no disabilities (87.55%, n = 3644), and approximately two-thirds (62.73%) were enrolled in self-employed health insurance. Furthermore, the majority of participants (91.64%, n = 3814) did not receive long-term care insurance. The mean annual inpatient and outpatient visits was 1.06 (range: 0–32 visits), and the average length of hospital stay was 2.03 days (range: 0–180 days).

### 3.2. Changes in Medical Expenditures Before and After MCI Diagnosis

**Figure 2:**
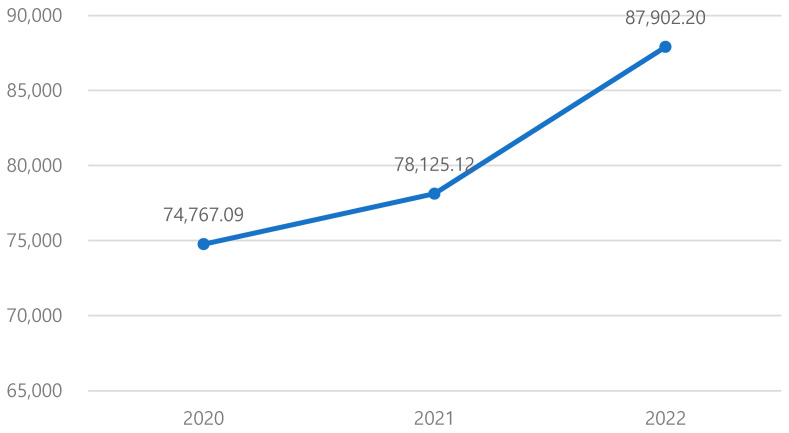
Comparison of average annual medical expenditures before and after MCI diagnosis (unit: KRW). (MCI: Mild Cognitive Impairment.)

The average annual total medical expenditure showed a significant increase following the diagnosis of MCI (Table 2) (Figure 2). Medical expenditures increased incrementally after the diagnosis of MCI, showing a year-on-year rise of 4.49% immediately following diagnosis (2021) compared to the pre-diagnosis period, and accelerating to a 12.51% increase in the post-diagnosis (2022) compared to diagnosed period.

**Figure 3:**
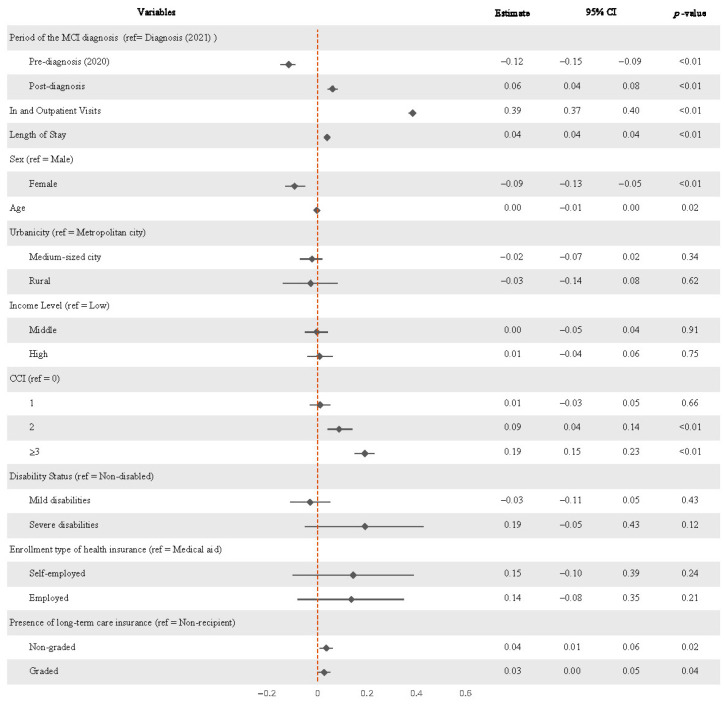
Forest Plot of the change in annual medical expenditures before and after MCI diagnosis. (MCI: Mild Cognitive Impairment; CCI: Charlson Comorbidity Index).

Significant changes in total medical expenditures following the diagnosis of MCI were observed in Table 3 and visualized in the Forest plot (Figure 3). Compared with the diagnosis year, expenditure significantly decreased in the year before diagnosis (β = −0.117, *p* < 0.01) approximately −11.04% but increased in the year following diagnosis by about 6% (β = 0.061, *p* < 0.01).

Several factors were significantly associated with higher medical expenditures. These included a longer length of hospital stay (β = 0.039, *p* < 0.01), a greater number of outpatient visits (β = 0.385, *p* < 0.01), enrollment in long-term care insurance—both non-graded (β = 0.035, *p* = 0.02) and graded (β = 0.027, *p* = 0.04)—as well as higher Charlson Comorbidity Index (CCI) scores (CCI = 2: β = 0.088, *p* = 0.04; CCI ≥ 3: β = 0.192, *p* < 0.01). In contrast, female sex (β = −0.093, *p* < 0.01) and older age (β = −0.003, *p* = 0.02) were associated with reduced healthcare expenditures.

The winsorized total expenditure was similar as the main analysis as shown in Supplementary Table S1.

### 3.3. Changes in Medical Expenditures Before and After MCI Diagnosis, Stratified by Sex

Significant changes in total medical expenditures following the diagnosis of MCI were also observed among both male and female patients (Table 4 and Table 5).

For males, compared with the diagnosis year, expenditures significantly decreased in the year prior to diagnosis (β = −0.142, *p* < 0.01) but increased in the year following diagnosis (β = 0.053, *p* = 0.02).

For females, a similar pattern was observed, with expenditures significantly decreasing before diagnosis (β = −0.104, *p* < 0.01), and increasing again in the year after diagnosis (β = 0.063, *p* < 0.01).

## 4. Discussion

This study analyzed changes in healthcare expenditures associated with the diagnosis of MCI, utilizing data from NHIS–NHID. The results revealed a noticeable rise in healthcare expenditures after MCI diagnosis, consistent with previous evidence indicating greater financial demands due to more frequent use of healthcare services [5,6,14,15]. In particular, longer hospital stays and more outpatient visits appeared to drive much of this increase—likely reflecting the challenges of managing chronic illnesses that worsen with cognitive decline [2,4,9,15,16]. People living with MCI often face difficulties keeping up with medications or appointments, which may be associated with greater dependence on healthcare [2,5,6].

Through descriptive statistics, we found that after an MCI diagnosis, total medical expenditures increased by 4.49% in the first post-diagnosis year (2021) and by 12.51% in the second (2022) relative to the pre-diagnosis year. This trajectory aligns with internationally observed studies showing increased expenditures after MCI diagnosis [6], thereby strengthening external validity to our results and highlighting clinically relevant rises in healthcare utilization. For example, a U.S. cohort study of individuals with MCI reported expenditures of $13,691 in the year before diagnosis, rising to $20,386 in the first post-diagnosis year, which is about 48.9% increase [6]. Taken together, our findings are consistent with internationally observed patterns of elevated cost after the diagnosis.

Regression analysis also identified the year of diagnosis as a critical turning point in spending. While medical expenditures decreased by 11.04% in the year prior to diagnosis (β = −0.117, *p* < 0.01), they increased by 6% in the subsequent year (β = 0.061, *p* < 0.01), demonstrating a statistically significant shift in cost trajectories.

Interestingly, the analysis found that female and older adults tended to have lower medical costs, a result that does not fully align with prior studies [1,3,7,8,10,17]. Cultural and systemic differences in the Korean healthcare context may help explain this variation [3,10,17]. For example, older individuals might be more accustomed to managing long-term conditions or may reduce their use of services due to financial concerns [1,7,8]. Some evidence also suggests that Korean seniors often use lower-cost preventive services—such as screenings or vaccinations—rather than more expensive specialist care, which could contribute to reduced costs overall [18,19]. Likewise, female might be more proactive about preventive health and may rely more on informal support networks, which could lower their need for costly medical service. Further research should explore these behavioral and economic factors more deeply

The study also observed that people enrolled in LTCI and those with multiple chronic conditions spent considerably more on healthcare. This aligns with the reality that complex cases demand more intensive management and resources [7,9,20,21]. Patients juggling several conditions typically require more frequent care, underscoring the need for tailored treatment approaches to help manage both health outcomes and costs [7,20,21].

Healthcare expenses also varied by region. People living in larger cities tended to spend more, which could reflect better access to hospitals and services [10,22]. This suggests that regional policies may be needed to reduce gaps in access and promote more balanced use of healthcare resources.

Income also appeared to play a role—those in higher income brackets tended to spend more, possibly due to greater access to a wider range of care options [16,22]. These differences emphasize the importance of designing policies that make healthcare more affordable and accessible, especially for those with fewer resources.

When comparing across types of insurance—such as medical aid, self-employed, and employer-sponsored plans—differences in coverage and cost-sharing could partly explain variations in spending [10,22]. Targeted policy changes could help reduce these insurance-based inequalities.

Further stratified analysis revealed sex-specific cost determinants. Among male, residing in mid-sized cities was associated with lower medical costs (β = −0.075, *p* = 0.03), whereas among female, healthcare costs decreased with increasing age (β = −0.003, *p* = 0.02) and increased significantly when CCI was ≥2. These differences may be rooted in gender-based variations in health-seeking behavior, accessibility, or informal care dynamics.

The presence of multimorbidity—particularly when CCI ≥ 3—was a consistent and strong predictor of elevated costs, with β coefficients ranging from 0.183 to 0.237. This suggests that total healthcare expenditures are more closely tied to patients’ overall clinical complexity than to cognitive decline alone.

Given the substantial post-diagnosis increase in outpatient service use, strengthening community-based care and early detection programs for at-risk groups could help reduce avoidable costs. Additionally, the heterogeneity of cost drivers across demographic subgroups underscores the importance of gender- and region-specific health policy planning. Moreover, disparities by income, insurance type, and geography point to systemic inequalities in healthcare access. These disparities often intersect with differences by age and gender, compounding barriers to care for certain populations. To address these compounded challenges, policy interventions should be explicitly designed with consideration of age and gender to improve access in underserved populations, address financial barriers, and ensure that preventive care is both accessible and affordable. In turn, such targeted policy actions are essential to enhance equity and efficiency, particularly in an aging society.

It is worth noting that while this study used large-scale national data, it focused only on direct medical costs. It did not include indirect burdens such as informal caregiving, lost productivity, or psychosocial strain, which likely underestimates the full societal impact of MCI. Moreover, claims-based diagnoses may not reflect clinical accuracy, and disease progression to dementia was not captured. Therefore, future studies should aim to include these aspects for a more complete understanding of MCI’s impact. For example, studies should explicitly incorporate indirect cost components, such as unpaid caregiving time, caregiver employment disruption, and productivity losses, to provide a more comprehensive and accurate understanding of MCI’s societal and economic implications. Further analysis is also needed to explore how costs evolve as the condition progresses.

## 5. Conclusions

The data in this study revealed a clear rise in healthcare spending after individuals were diagnosed with MCI. Those enrolled in long-term care insurance programs and patients with multiple health conditions saw particularly steep increases. Spending patterns also differed depending on where people lived, their income, and the type of insurance they had.

Notably, gender-specific cost drivers were identified: older females tended to incur lower healthcare expenditures, whereas men living in mid-sized cities experienced regionally influenced spending patterns. These differences suggest that healthcare utilization may be shaped by behavioral, socioeconomic, and regional factors.

These findings diverge from prior international studies, which have often reported higher healthcare costs among older adults and females. This discrepancy may reflect unique characteristics of Korea’s healthcare system, including cultural attitudes toward care-seeking and financial barriers to service utilization.

These findings highlight the importance of early and personalized care strategies to mitigate the rising healthcare costs associated with MCI. Policymakers should prioritize enhancing access in underserved regions, expanding financial support for low-income groups, and refining insurance benefits to address service gaps. Interventions that consider differences in gender and age may further enhance care effectiveness. To reduce healthcare disparities, equity-based policies must be implemented to address variations in access and affordability across income levels, age groups, genders, and geographic areas.

Moreover, from a clinical perspective, the observed sex-specific cost pattern could support risk stratification and care planning for patients with MCI. For instance, clinicians could prioritize comorbidity optimization and early cognitive screening for men, considering relevant influencing factors, particularly for those living in regions with higher healthcare expenditures. In contrast, for older women, strategies could address the factors influencing healthcare costs, with a focus on reinforcing preventive care pathways and managing polypharmacy. Such tailored approaches may enhance patient outcomes, optimize resource utilization, and reduce avoidable expenditures in routine practice.

Going forward, research should aim to include both direct and indirect costs, such as caregiver time and productivity losses, to provide more a comprehensive assessment of MCI’s economic impact. Studying how costs change depending on a patient’s condition over time—and how those patterns differ by demographic group—will be essential for making better policy decisions and ensuring efficient allocation of healthcare resources. Ultimately, better-informed strategies may lead to improved care and reduced burden for individuals living with MCI and their families. Without deliberate efforts to close these gaps, the economic burden of MCI will continue to disproportionately affect vulnerable populations.

## Figures and Tables

**Table 1:** Characteristics of the study population at the time of MCI diagnosis.

Variables		N/Mean + S.D.	%
In and Outpatient Visits		1.06 ± 1.02	
Length of Stay		2.03 ± 6.77	
Sex	Male	1380	33.16
	Female	2782	66.84
Age		70.59 ± 7.53	
Urbanicity	Metropolitan city	1747	41.98
	Medium-sized city	1913	45.96
	Rural	502	12.06
Income Level	Low	2011	48.32
	Middle	950	22.83
	High	1201	28.86
CCI	0	1185	28.47
	1	677	16.27
	2	630	15.14
	≥3	1670	40.12
Disability Status	Non-disabled	3644	87.55
	Mild disabilities	415	9.97
	Severe disabilities	103	2.47
Enrollment type of health insurance	Medical aid	1322	31.76
	Self-employed	2611	62.73
	Employed	229	5.5
Presence of long-term care insurance	Non-recipient	3814	91.64
	Non-graded	257	6.17
	Graded	91	2.19

S.D.: Standard Error; MCI: Mild Cognitive Impairment; CCI: Charlson Comorbidity Index.

**Table 2:** Comparison of average annual medical expenditures before and after MCI diagnosis.

Variables		Mean (KRW)	S.D	Max (KRW)	Min (KRW)
Period of the MCI diagnosis	Diagnosis (2021)	78,125.12 ($57.61)	126,093.39	5460 ($4.03)	2,705,468.33 ($1995.18)
	Pre-diagnosis (2020)	74,767.09 ($55.13)	174,991.36	4670 ($3.44)	6,183,749.09 ($4560.29)
	Post-diagnosis (2022)	87,902.2 ($64.82)	171,618.41	8160 ($6.02)	3,691,224.29 ($2722.14)

(S.D.: Standard Error; MCI: Mild Cognitive Impairment.) The Korean won (KRW) was converted to USD by applying an exchange rate of 1356 KRW per 1 USD (as of 2 July 2025).

**Table 3:** Comparison of medical expenditures before and after MCI diagnosis.

Variables		Estimate	95% CI		*p*-Value
			LL	UL	
Period of the MCI diagnosis	Diagnosis (2021)	Ref.			
	Pre-diagnosis (2020)	−0.117	−0.15	−0.09	<0.01
	Post-diagnosis	0.061	0.04	0.08	<0.01
In and Outpatient Visits		0.385	0.37	0.40	<0.01
Length of Stay		0.039	0.03	0.04	<0.01
Sex	Male	Ref.			
	Female	−0.093	−0.13	−0.05	<0.01
Age		−0.003	−0.01	−0.00	0.02
Urbanicity	Metropolitan city	Ref.			
	Medium-sized city	−0.023	−0.07	0.02	0.34
	Rural	−0.028	−0.14	0.08	0.62
Income Level	Low	Ref.			
	Middle	−0.003	−0.05	0.04	0.91
	High	0.008	−0.04	0.06	0.75
CCI	0	Ref.			
	1	0.010	−0.03	0.05	0.66
	2	0.088	0.04	0.14	<0.01
	≥3	0.192	0.15	0.23	<0.01
Disability Status	Non-disabled	Ref.			
	Mild disabilities	−0.030	−0.11	0.05	0.43
	Severe disabilities	0.192	−0.05	0.43	0.12
Enrollment type of health insurance	Medical aid	Ref.			
	Self-employed	0.145	−0.10	0.39	0.24
	Employed	0.137	−0.08	0.35	0.21
Presence of long-term care insurance	Non-recipient	Ref.			
	Non-graded	0.035	0.01	0.06	0.02
	Graded	0.027	0.00	0.05	0.04

CI: Confidence Interval; LL: Lower Limit; UL: Upper Limit; MCI: Mild Cognitive Impairment; CCI: Charlson Comorbidity Index.

**Table 4:** Comparison of medical expenditures before and after MCI diagnosis among males.

Variables		Estimate	95% CI		*p*-Value
			LL	UL	
Period of the MCI diagnosis	Diagnosis (2021)	Ref.			
	Pre-diagnosis (2020)	−0.142	−0.19	−0.09	<0.01
	Post-diagnosis	0.053	0.01	0.10	0.02
In and Outpatient Visits		0.385	0.407	0.39	<0.01
Length of Stay		0.039	0.042	0.03	<0.01
Age		−0.003	−0.002	−0.01	0.51
Urbanicity	Metropolitan city	Ref.			
	Medium-sized city	−0.075	−0.14	−0.01	0.03
	Rural	−0.069	−0.17	0.04	0.20
Income Level	Low	Ref.			
	Middle	0.016	−0.08	0.11	0.74
	High	−0.028	−0.11	0.06	0.52
CCI	0	Ref.			
	1	−0.013	−0.09	0.07	0.76
	2	0.076	−0.02	0.17	0.12
	≥3	0.237	0.15	0.32	<0.01
Disability Status	Non-disabled	Ref.			
	Mild disabilities	−0.012	−0.15	0.13	0.87
	Severe disabilities	0.251	−0.10	0.60	0.16
Enrollment type of health insurance	Medical aid	Ref.			
	Self-employed	0.105	−0.09	0.3	0.29
	Employed	0.133	−0.05	0.31	0.15
Presence of long-term care insurance	Non-recipient	Ref.			
	Non-graded	0.059	−0.01	0.12	0.08
	Graded	0.062	0.00	0.12	0.05

CI: Confidence Interval; LL: Lower Limit; UL: Upper Limit; MCI: Mild Cognitive Impairment; CCI: Charlson Comorbidity Index.

**Table 5:** Comparison of medical expenditures before and after MCI diagnosis among females.

Variables		Estimate	95% CI		*p*-Value
			LL	UL	
Period of the MCI diagnosis	Diagnosis (2021)	Ref.			
	Pre-diagnosis (2020)	−0.104	−0.14	−0.07	<0.01
	Post-diagnosis	0.063	0.04	0.09	<0.01
In and Outpatient Visits		0.369	0.35	0.39	<0.01
Length of Stay		0.037	0.03	0.04	<0.01
Age		−0.003	−0.01	0.00	0.02
Urbanicity	Metropolitan city				
	Medium-sized city	0.005	−0.08	0.09	0.91
	Rural	0.039	−0.17	0.25	0.72
Income Level	Low	Ref.			
	Middle	−0.003	−0.05	0.05	0.92
	High	0.036	−0.03	0.1	0.26
CCI	0	Ref.			
	1	0.026	−0.03	0.08	0.33
	2	0.105	0.05	0.16	<0.01
	≥3	0.183	0.13	0.23	<0.01
Disability Status	Non-disabled	Ref.			
	Mild disabilities	−0.044	−0.12	0.03	0.24
	Severe disabilities	−0.038	−0.15	0.07	0.50
Enrollment type of health insurance	Medical aid	Ref.			
	Self-employed	0.204	−0.19	0.6	0.31
	Employed	0.183	−0.18	0.54	0.32
Presence of long-term care insurance	Non-recipient	Ref.			
	Non-graded	0.025	−0.01	0.06	0.20
	Graded	0.018	−0.01	0.05	0.23

CI: Confidence Interval; LL: Lower Limit; UL: Upper Limit; MCI: Mild Cognitive Impairment; CCI: Charlson Comorbidity Index.

## Data Availability

The data used in this study are from the National Health Information Database (NHID), which is maintained by the Korean National Health Insurance Service (NHIS). Access to the data is restricted to researchers who have been approved by the NHIS, and the data is not publicly available.

## Supplementary Materials

The following supporting information can be downloaded at: https://www.mdpi.com/article/10.3390/healthcare13162076/s1, Table S1: Comparison of medical expenditures before and after MCI diagnosis after adjusting outlier.

## Abbreviations

MCI	Mild Cognitive Impairment
NHIS	National Health Insurance Service
NHID	National Health Information Database
ICD-10	International Classification of Diseases, 10th Revision
CCI	Charlson Comorbidity Index
LTCI	Long-Term Care Insurance
GEE	Generalized Estimating Equation
