# Supplementary material for: Healthcare Spending Before and After Mild Cognitive Impairment Diagnosis: Evidence from the NHIS–NHID in Korea
Source: Healthcare (Basel). 2025 Aug 21;13(16):2076. doi: 10.3390/healthcare13162076 (PMC12386116; doi:10.3390/healthcare13162076)
Supplement: Supplementary file 1 [file healthcare-13-02076-s001.zip › healthcare-3774766-supplementary.pdf]

Supplementary Table S1. Comparison of medical expenditures before and after MCI diagnosis after adjusting outlier.

Variables		Estimate	95% CI		*p*-value
			LL	UL	
Period of the MCI diagnosis	Diagnosis (2021)	Ref.			
	Pre-diagnosis (2020)	−0.117	−0.15	−0.09	<0.01
	Post-diagnosis	0.062	0.04	0.09	<0.01
In and Outpatient Visits		0.385	0.37	0.36	<0.01
Length of Stay		0.039	0.04	0.03	<0.01
Sex	Male	Ref.			
	Female	−0.091	−0.13	−0.05	<0.01
Age		−0.003	−0.00	−0.01	0.02
Urbanicity	Metropolitan city	Ref.			
	Medium-sized city	−0.027	−0.07	0.02	0.24
	Rural	−0.030	−0.14	0.08	0.60
Income Level	Low	Ref.			
	Middle	−0.002	−0.05	0.04	0.93
	High	0.013	−0.04	0.06	0.62
CCI	0	Ref.			
	1	0.012	−0.03	0.05	0.57
	2	0.090	0.04	0.14	<0.01
	≥3	0.193	0.15	0.23	<0.01
Disability Status	Non-disabled	Ref.			
	Mild disabilities	−0.031	−0.11	0.04	0.41
	Severe disabilities	0.195	−0.05	0.44	0.11
Enrollment type of health insurance	Medical aid	Ref.			
	Self-employed	0.148	−0.09	0.39	0.23
	Employed	0.140	−0.08	0.36	0.21
Presence of long-term care insurance	Non-recipient	Ref.			
	Non-graded	0.033	0.00	0.06	0.03
	Graded	0.026	0.00	0.02	<0.05

CI: Confidence Interval; LL: Lower Limit; UL: Upper Limit; CCI: Charlson Comorbidity Index
